# Mental health and help-seeking in Czech sexual minorities: a nationally representative cross-sectional study

**DOI:** 10.1017/S2045796024000210

**Published:** 2024-03-21

**Authors:** Michal Pitoňák, Libor Potočár, Tomáš Formánek

**Affiliations:** 1 Department of Public Mental Health, National Institute of Mental Health, Klecany, Czechia; 2 PROMENTA Research Center, Department of Psychology, University of Oslo, Oslo, Norway; 3 Department of Psychiatry, University of Cambridge, Cambridge, UK

**Keywords:** mental disorders, population survey, prevalence rate, probability sample, sexual minority, sexual orientation, suicidality, treatment gap

## Abstract

**Aims:**

The mental health of sexual minority (SM) individuals remains overlooked and understudied in Czechia. We aimed to estimate (1) the prevalence rate and (2) the relative risk of common mental disorders and (3) the mental distress severity among the Czech SM people compared with the heterosexual population. In addition, we aimed to investigate help-seeking for mental disorders in SM people.

**Methods:**

We used data from a cross-sectional, nationally representative survey of Czech community-dwelling adults, consisting of 3063 respondents (response rate = 58.62%). We used the Mini-International Neuropsychiatric Interview to assess the presence of mental disorders. In individuals scoring positively, we established help-seeking in the past 12 months. We assessed symptom severity using the 9-item Patient Health Questionnaire and the 7-item Generalized Anxiety Disorder scale. We computed the prevalence of mental disorders and the treatment gap with 95% confidence intervals. To assess the risk of having a mental disorder, we used binary logistic regression.

**Results:**

We demonstrated that the prevalence of current mental disorders was 18.85% (17.43–20.28), 52.27% (36.91–67.63), 33.33% (19.5–47.17) and 25.93% (13.85–38) in heterosexual, gay or lesbian, bisexual and more sexually diverse individuals, respectively. Suicidal thoughts and behaviours were present in 5.73% (4.88–6.57), 25.00% (11.68–38.32), 22.92% (10.58–35.25) and 11.11% (2.45–19.77) of heterosexual, gay or lesbian, bisexual and more sexually diverse individuals, respectively. After confounder adjustment, gay or lesbian individuals were more likely to have at least one current mental disorder compared with heterosexual counterparts (odds ratio = 3.51; 1.83–6.76). For bisexual and sexually more diverse individuals, the results were consistent with a null effect (1.85; 0.96–3.45 and 0.89; 0.42–1.73). The mean depression symptom severity was 2.96 (2.81–3.11) in heterosexual people and 4.68 (2.95–6.42), 7.12 (5.07–9.18) and 5.17 (3.38–6.95) in gay or lesbian, bisexual and more sexually diverse individuals, respectively. The mean anxiety symptom severity was 1.97 (1.85–2.08) in heterosexual people and 3.5 (1.98–5.02), 4.63 (3.05–6.2) and 3.7 (2.29–5.11) in gay or lesbian, bisexual and more sexually diverse individuals, respectively. We demonstrated broadly consistent levels of treatment gap in heterosexual and SM individuals scoring positively for at least one current mental disorder (82.91%; 79.5–85.96 vs. 81.13%; 68.03–90.56).

**Conclusions:**

We provide evidence that SM people in Czechia have substantially worse mental health outcomes than their heterosexual counterparts. Systemic changes are imperative to provide not only better and more sensitive care to SM individuals but also to address structural stigma contributing to these health disparities.

## Introduction

Contemporary research consistently reveals that sexual minority (SM) individuals are more likely to experience worsened mental health outcomes compared with their heterosexual counterparts, with the most documented disparities encompassing a higher occurrence of depression and anxiety disorders (Pachankis *et al.*, 2021; Ploderl and Tremblay, 2015), markedly elevated rates of suicidal thoughts and behaviours (Yildiz, 2018) and increased risk of substance use (Marshal *et al.*, 2008; Schuler and Collins, 2020). Over time, it is also becoming evident that there are disparities among SM individuals, with certain groups, such as bisexual individuals, displaying more pronounced mental health challenges (Pakula *et al.*, 2016; Ross *et al.*, 2018) and higher substance use rates (Schuler and Collins, 2020) than both their heterosexual and gay and lesbian counterparts.

Previous research building on frameworks such as the minority stress theory (Brooks, 1981; Meyer, 2003) or related models, such as the rejection sensitivity model (Feinstein, 2020), the health equity promotion model (Fredriksen-Goldsen *et al.*, 2014) or the psychological mediation framework (Hatzenbuehler, 2009), has substantiated that these mental health disparities are largely due to societal stigma, discrimination and lack of acceptance experienced by SM individuals (Dürrbaum and Sattler, 2020; Pachankis and Bränström, 2018; Zeeman *et al.*, 2019), clearly denouncing the historically dominant pathologizing perspectives (Drescher, 2015).

Nonetheless, the research focusing on the mental health of SM individuals is not evenly distributed, with a systematic review that encompassed 199 studies revealing that 76% of these investigations were carried out in the US or Canada (Ploderl and Tremblay, 2015). This research remains comparatively scarce in the region of Central and Eastern Europe (CEE), including Czechia, although notable studies exist (Chumakov *et al.*, 2023; Cisek and Rogowska, 2023; Iniewicz *et al.*, 2017; Kardasz *et al.*, 2023; Kranz *et al.*, 2023; Pitoňák *et al.*, 2023a; Šević *et al.*, 2016; Stojanovski *et al.*, 2022). This is particularly noteworthy when we consider that the absence of sexual orientation questions in public health surveys constitutes a fundamental barrier limiting the generation of primary data on the mental health of SM populations (Bränström *et al.*, 2019), thus allowing policymakers in the CEE region to overlook these people and their needs.

This oversight can be, then, viewed in light of structural stigma that encompasses laws and policies that deny or fail to protect equal rights for SM people, alongside prejudicial population attitudes (Hatzenbuehler, 2016). Even though previous research has demonstrated a link between structural stigma and the mental health of SM people, including in CEE countries (Bränström *et al.*, 2022; Pachankis *et al.*, 2021), there is a strong reluctance among policymakers to implement legal changes that would have a positive impact on SMs. This includes the passing of marriage equality legislation and the recognition of same-sex families through the allowance of joint adoptions by same-sex couples (Pitoňák, 2023). Indeed, in Czechia, no substantial legal change has occurred since the introduction of registered partnership in 2006, 18 years ago.

The first Czech study on SM mental health demonstrated that all SM subgroups had significantly higher levels of psychological distress relative to the general population, with the rates being higher in bisexual than in gay and lesbian respondents (Pitoňák *et al.*, 2023a). However, there were some important limitations in that study, including the use of non-probability sampling and a non-specific mental distress self-report scale to assess mental health outcomes (Pitoňák *et al.*, 2023a). Moreover, while existing research has demonstrated that SM people may face additional barriers when seeking help for their mental health (McDermott *et al.*, 2018; Spengler *et al.*, 2023), no evidence on help-seeking behaviour in Czech SMs is available.

We are convinced that providing robust evidence on mental health and associated needs may be one of the key steps needed to overcome the profound oversight that SM people experience in Czechia. In the present study, we aimed to use data from the first Czech nationally representative probability survey that included sexual identity and mental health measures to provide robust evidence on mental health and associated needs of Czech SM individuals. Specifically, we aimed to estimate (1) the prevalence rate and (2) the relative risk of common mental disorders and (3) the mental distress severity among the SM individuals compared with the heterosexual population. In addition, we aimed to investigate the help-seeking behaviours of SM people in Czechia. We hypothesized that SM individuals would show an increased risk of anxiety disorders, depression, suicidal thoughts and behaviours, and an increased mental distress severity compared with the heterosexual population. We hypothesized that the most affected SM group would be bisexual individuals, followed by lesbian or gay individuals (i.e., bisexual > lesbian or gay > heterosexual).

## Methods

The research questions and the analytical plan were pre-registered at Open Science Framework before data analyses started (Pitoňák *et al.*, 2023b). Any deviations from the analytical plan are described in Supplementary Methods.

### Data

We analysed data from a cross-sectional survey of Czech community-dwelling adults that was conducted by a professional data collection agency in November and December 2022. Three different data collection methodologies were employed: (1) household probability sampling and computer-assisted personal interviewing, (2) panel sampling and computer-assisted telephone interviewing and (3) panel sampling and computer-assisted online interviewing. For personal interviewing, a two-stage sampling method was employed. The procedure involved randomly selecting a sample of voting districts and a random starting address in each of these. In multi-apartment dwellings, the interviewers were asked to choose the fourth apartment when counting from the top. Then, in single‐member households, individuals aged 18 or more years were eligible for interviewing, whereas in multi-member households, the person with their birthday closest to the date of the interviewer’s visit was asked to participate. In the online and telephone interviewing modes, the procedure consisted of randomly emailing or telephoning individuals present in a panel of a professional data collection agency. In the initial step, strata by age, sex, level of education and region of residence were created, and the required number of respondents for each stratum was established. Then, unique identifiers corresponding to this were drawn from the panel, and the chosen individuals were contacted to participate in the study. If the chosen person declined to participate following repeated reminders, then another person with the same target sociodemographic characteristic was contacted. The procedure was repeated until the required number of respondents for each stratum was reached. The dataset consists of 3063 (response rate [RR] = 58.62%), 3248 (RR = 29.75%) and 1000 (RR = 8.97%) respondents who completed the personal, online and telephone versions of the survey, respectively. The samples were representative of the Czech adult population in terms of age, sex, education and region of residence. Additional technical parameters of the data are provided elsewhere (Potočár *et al.*, 2024).

To increase the confidence in the validity of our results, we restricted the main analyses to data from household probability sampling and personal interviewing, which represents a golden standard of mental health assessment. We used data from panel samples interviewed online and by telephone in sensitivity analyses. All respondents provided informed consent. The study was approved by the Ethics Committee of the National Institute of Mental Health (registration number: 173/21).

### Measures

To assess sexual orientation, we employed a self-identification approach. Respondents were asked the question ‘Which option best describes your sexual orientation?’, with the response options being ‘heterosexual’, ‘gay’, ‘lesbian’, ‘bisexual’ and ‘other’ (Sell, 2007). To prevent unnecessary ‘othering’, we coded respondents who selected ‘other’ as being ‘more sexually diverse’. To assess sex, the respondents were asked to report ‘Sex (assigned at birth)’, with response options being ‘male’ and ‘female’, and in terms of gender identity, the respondents were asked ‘How would you describe yourself?’ and could choose from ‘man’, ‘woman’, ‘transgender’, ‘I do not identify neither as man, woman nor transgender’ (Badgett *et al.*, 2014). To facilitate the disclosure of authentic identities, respondents in the personal and telephone interviewing modes were not required to say out loud the entire response option (e.g., ‘bisexual’), instead, the provision of the letter denoting the response option was sufficient (e.g., ‘d’).

Then, to assess the occurrence of mental disorders, we used the 5th version of the Mini-International Neuropsychiatric Interview (M.I.N.I.), a structured psycho-diagnostic interview (Sheehan *et al.*, 1998). The diagnostic criteria in M.I.N.I. correspond to the 4th edition of the Diagnostic and Statistical Manual of Mental Disorders and the 10th version of the International Classification of Diseases (Sheehan *et al.*, 1998). The survey collected data on a subset of M.I.N.I. modules, including (1) a major depressive episode (MDE), (2) anxiety disorders (panic disorder, generalized anxiety disorder, agoraphobia, social phobia and posttraumatic stress disorder), (3) alcohol use disorders (AUDs; alcohol dependence and alcohol abuse) and (4) suicidal thoughts and behaviours.

In individuals who scored positively for mental disorders on M.I.N.I., we established whether they had sought medical or other professional help due to their mental health in the past 12 months. The list of medical or other professionals consisted of (1) psychiatrists, (2) psychologists, (3) general practitioners, (4) crisis interventions and (5) online therapists or online therapeutic platforms. We considered not seeking help from any of the above professionals as being indicative of a treatment gap for mental disorders.

We assessed the depression- and anxiety-related symptom severity using the 9-item Patient Health Questionnaire (PHQ-9) (Kroenke *et al.*, 2001) and the 7-item Generalized Anxiety Disorder scale (GAD-7) (Spitzer *et al.*, 2006). We computed composite scores by summing up all individual items. The values on PHQ-9 can, then, range from 0 to 27, whereas on GAD-7 from 0 to 21: higher values indicate higher symptom severity on both instruments. On both instruments, scores ≥5 and ≥10 indicate mild and moderate symptomatology, respectively (Kroenke *et al.*, 2001; Spitzer *et al.*, 2006).

Finally, we considered several sociodemographic variables as confounders, including age, gender, work status, level of education, income level, relationship status (married or lives with a partner/spouse, married but separated or in a relationship but living apart, widowed, divorced and single) and size of the region of residence. We merged respondents who were married with those who were otherwise partnered/spoused into one category to account for uneven institutional conditions that prevent SM people from becoming married in Czechia.

### Statistical analyses

We computed the prevalence rate of (1) any mental disorder, (2) MDE, (3) anxiety disorders, (4) AUDs and (5) suicidal thoughts and behaviours with 95% confidence intervals (95% CIs), stratified by respondents’ sexual orientation identity (i.e., heterosexual, gay or lesbian, bisexual and more sexually diverse).

To assess the risk of having a mental disorder, we used binary logistic regression with sexual orientation identity as exposure while controlling for a set of potential confounders associated with mental health (i.e., age, gender, education, work status, income level, relationship status and size of the region of residence). The reference group was heterosexual individuals. To assess the differences between SM subgroups, we compared bisexual and more sexually diverse individuals with gay or lesbian individuals (reference group) while adjusting for the same set of confounders as specified above.

Then, to analyse depression- and anxiety-related symptom severity, we used linear regression models with PHQ-9 and GAD-7 composite scores as outcomes, sexual orientation identity as exposure and heterosexual individuals as reference group while adjusting for the same set of confounders as specified above. To assess the differences between SM subgroups, we compared bisexual and more sexually diverse individuals with gay or lesbian individuals (reference group) while adjusting for above confounders. We log-transformed the PHQ-9 and GAD-7 scores before their inclusion into the models and estimated robust (sandwich) standard errors.

For those who scored positively for (1) any mental disorder, (2) MDE, (3) anxiety disorders, (4) AUDs and (5) suicidal thoughts and behaviours, we estimated the treatment gap stratified by SM status (gay or lesbian or bisexual or more sexually diverse vs. heterosexual individuals).

For the prevalence of mental disorders and treatment gap prevalence, we computed 95% CIs using the delta method. If a subgroup had ≤5 individuals, we calculated the exact binomial (Clopper–Pearson) 95% CIs. We conducted all data preprocessing steps and statistical analyses in R statistical programming language (version 4.2.2) (R Core Team, 2024).

### Sensitivity analyses

We assessed the prevalence rates and relative risk of mental disorders, the mental distress severity, treatment gap and help-seeking behaviour using data from panel samples, interviewed online and by telephone. The analytical approach was analogous to the one used in the main analysis. Additionally, in regression models, we included the mode of interviewing (telephone vs. online) in the set of confounders. Based on the substantial differences between results obtained by different data collection methodologies that we demonstrated previously (Potočár *et al.*, 2024), we applied a cautious approach to panel data and used them only to assess whether mental health outcomes in SM people were broadly consistent with the results from the main analysis.

## Results

### Sample

The sample consisted of 2917 (95.2%) heterosexual, 44 (1.44%) gay or lesbian, 48 (1.57%) bisexual and 54 (1.76%) more sexually diverse individuals. Gay or lesbian (mean = 34.64; standard deviation = 12.10) and bisexual individuals (34.81; 15.56) tended to be younger than heterosexual individuals (49.86; 16.76). Compared with heterosexual people, a higher proportion of gay and lesbian individuals had a university-level education (29.55% vs. 18.17%), was employed (65.91% vs. 50.74%), had the highest income level (18.18% vs. 4.05%) and lived in large cities with more than 100,000 inhabitants (40.91% vs. 21.43%). For more detailed descriptive statistics, see Table 1.

**Table 1.** Descriptive statistics of the sample

	Sexual orientation identity			
	Heterosexual	Gay or lesbian	Bisexual	More sexually diverse
Sample size, *n* (%)	2917 (95.2%)	44 (1.44%)	48 (1.57%)	54 (1.76%)
Sex, *n* (%)				
Female	1587 (54.41)	19 (43.18)	33 (68.75)	32 (59.26)
Male	1330 (45.59)	25 (56.82)	15 (31.25)	22 (40.74)
Gender, *n* (%)				
Women	1584 (54.3)	18 (40.91)	32 (66.67)	30 (55.56)
Men	1332 (45.66)	24 (54.55)	15 (31.25)	19 (35.19)
Non-binary	0 (0)	2 (4.55)	0 (0)	5 (9.26)
Transgender	1 (0.03)	0 (0)	1 (2.08)	0 (0)
Age, mean (SD)	49.86 (16.76)	34.64 (12.1)	34.81 (15.56)	44.26 (18.08)
Relationship status, *n* (%)				
Married/in a relationship, living together	1896 (65)	11 (25)	24 (50)	27 (50)
Married/in a relationship, living apart	112 (3.84)	9 (20.45)	4 (8.33)	2 (3.7)
Single	319 (10.94)	21 (47.73)	14 (29.17)	15 (27.78)
Divorced	307 (10.52)	3 (6.82)	4 (8.33)	5 (9.26)
Widowed	283 (9.7)	0 (0)	2 (4.17)	5 (9.26)
Level of education, *n* (%)				
Primary	361 (12.38)	4 (9.09)	9 (18.75)	14 (25.93)
Lower secondary	990 (33.94)	9 (20.45)	13 (27.08)	19 (35.19)
Upper secondary	1036 (35.52)	18 (40.91)	19 (39.58)	14 (25.93)
University	530 (18.17)	13 (29.55)	7 (14.58)	7 (12.96)
Work status, *n* (%)				
Employed	1480 (50.74)	29 (65.91)	19 (39.58)	26 (48.15)
Unemployed	65 (2.23)	3 (6.82)	1 (2.08)	5 (9.26)
Self-employed	244 (8.36)	2 (4.55)	3 (6.25)	0 (0)
Student	126 (4.32)	8 (18.18)	10 (20.83)	3 (5.56)
Retired	755 (25.88)	1 (2.27)	3 (6.25)	11 (20.37)
Parental leave	131 (4.49)	0 (0)	9 (18.75)	3 (5.56)
Other	116 (3.98)	1 (2.27)	3 (6.25)	6 (11.11)
Income category, *n* (%)				
0−9k CZK	173 (5.93)	5 (11.36)	9 (18.75)	8 (14.81)
10−19k CZK	833 (28.56)	4 (9.09)	13 (27.08)	17 (31.48)
20−29k CZK	661 (22.66)	9 (20.45)	10 (20.83)	11 (20.37)
30−39k CZK	384 (13.16)	9 (20.45)	4 (8.33)	10 (18.52)
40−49k CZK	162 (5.55)	3 (6.82)	3 (6.25)	2 (3.7)
50+k CZK	118 (4.05)	8 (18.18)	0 (0)	0 (0)
Decided not to disclose	586 (20.09)	6 (13.64)	9 (18.75)	6 (11.11)
Size of the region of residence, *n* (%)				
>5000	1149 (39.39)	5 (11.36)	19 (39.58)	15 (27.78)
5000−19,999	519 (17.79)	12 (27.27)	12 (25)	13 (24.07)
20,000−99,999	624 (21.39)	9 (20.45)	8 (16.67)	8 (14.81)
100,000+	625 (21.43)	18 (40.91)	9 (18.75)	18 (33.33)

The results are expressed as either absolute numbers (*n*) with percent proportions (%) or averages with standard deviations (SD).

### Prevalence and relative risk of common mental disorders

**Figure 1.**
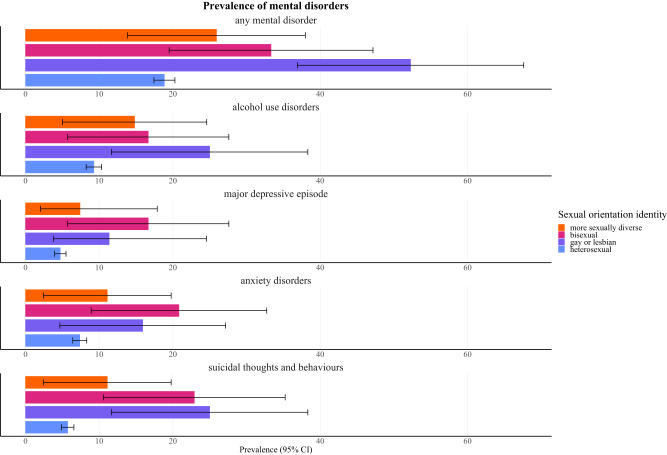
Prevalence of mental disorders per M.I.N.I.

We demonstrated that 18.85% (95% CI = 17.43–20.28) of heterosexual individuals met the criteria for any current mental disorder. In stark contrast, the prevalence rate for any current mental disorder was 52.27% (36.91–67.63) in gay or lesbian, 33.33% (19.5–47.17) in bisexual and 25.93% (13.85–38.00) in more sexually diverse individuals. The proportion of individuals scoring positively for current MDE was 4.73% (3.96–5.50) in heterosexual, 11.36% (3.79–24.56) in gay or lesbian, 16.67% (5.73–27.60) in bisexual and 7.41% (2.06–17.89) in more sexually diverse individuals. AUDs were present in 9.29% (8.24–10.34) of heterosexual, 25.00% (11.68–38.32) of gay or lesbian, 16.67% (5.73–27.60) of bisexual and 14.81% (5.03–24.60) of more sexually diverse individuals. The proportion of individuals that had an anxiety disorder was 7.37% (6.42–8.32) in heterosexual, 15.91% (4.66–27.16) in gay or lesbian, 20.83% (8.92–32.75) in bisexual and 11.11% (2.45–19.77) in more sexually diverse individuals. Considering suicidal thoughts and behaviours, 5.73% (4.88–6.57) of heterosexual, 25.00% (11.68–38.32) of gay or lesbian, 22.92% (10.58–35.25) of bisexual and 11.11% (2.45–19.77) of more sexually diverse individuals met the diagnostic criteria. The detailed results are provided in Fig. 1 and Supplementary Table S2.

The results are expressed as prevalence rates with 95% confidence intervals.

**Figure 2.**
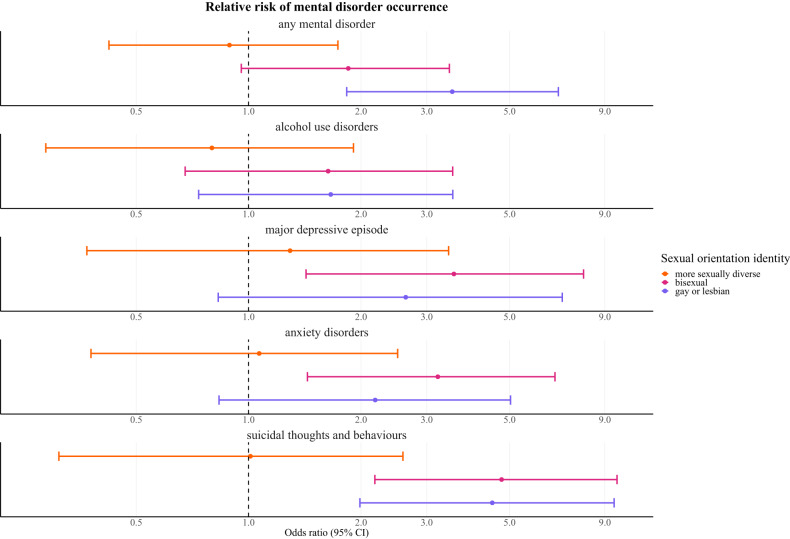
Relative risk of mental disorder occurrence per M.I.N.I.

After controlling for sociodemographic confounders, gay or lesbian individuals (odds ratio = 3.51; 95% CI = 1.83–6.76) had an elevated risk for fulfilling the criteria for having any mental disorder compared with heterosexual counterparts. For bisexual individuals, the 95% CI covered a range from a decreased to an increased risk (1.85; 0.96–3.45). We demonstrated increased risk for having a MDE and anxiety disorders in bisexual individuals (3.55; 1.42–7.89 and 3.21; 1.44–6.62), whereas in gay or lesbian people, the 95% CI covered a range from a decreased to an increased risk (2.64; 0.83–6.92 and 2.18; 0.83–5.03). For AUDs, 95% covered a range from a decreased to an increased risk in both gay or lesbian (1.66; 0.73–3.52) and bisexual (1.63; 0.68–3.52) individuals. We demonstrated an increased risk for having suicidal thoughts and behaviours in both gay or lesbian (4.49; 1.99–9.53) and bisexual (4.76; 2.18–9.70) individuals. In more sexually diverse individuals, the 95% CI covered a range from decreased to an increased risk for having any mental disorder (0.89; 0.42–1.73), AUDs (0.80; 0.29–1.91), MDE (1.29; 0.37–3.43), anxiety disorders (1.07; 0.38–2.51) and suicidal thoughts and behaviours (1.01; 0.31–2.59). The detailed results are provided in Fig. 2 and Supplementary Table S3.

The results are expressed as adjusted odds ratios with 95% confidence intervals. The models were adjusted for age, gender, education, work status, income level, relationship status and size of the region of residence. Heterosexual individuals were used as reference category.

In subgroup analyses with gay or lesbian individuals as reference group, more sexually diverse individuals had a decreased risk for having any mental disorder (0.25; 0.10–0.63) and suicidal thoughts and behaviours (0.23; 0.06–0.74). For other outcomes in more sexually diverse individuals as well as for each outcome in bisexual individuals, the results were consistent with a null effect. For detailed results, see Supplementary Table S3.

### Mental distress severity

**Figure 3.**
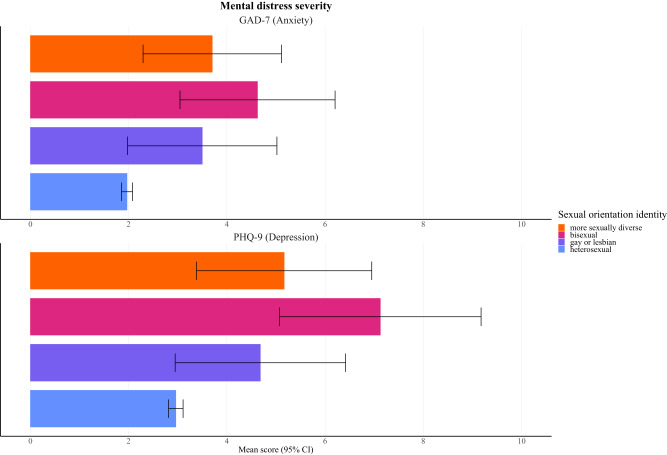
Mean PHQ-9 and GAD-7 scores

Heterosexual individuals had a mean depression symptom severity of 2.96 (95% CI = 2.81–3.11), whereas, in gay or lesbian, bisexual, and more sexually diverse individuals, it was 4.68 (2.95–6.42), 7.12 (5.07–9.18) and 5.17 (3.38–6.95), respectively. Considering anxiety, heterosexual people had a mean symptom severity of 1.97 (1.85–2.08), whereas, in gay or lesbian, bisexual, and more sexually diverse individuals, we detected 3.50 (1.98–5.02), 4.63 (3.05–6.20) and 3.70 (2.29–5.11), respectively. For detailed results, see Fig. 3 and Supplementary Table S4.

The results are expressed as averages with 95% confidence intervals.

**Figure 4.**
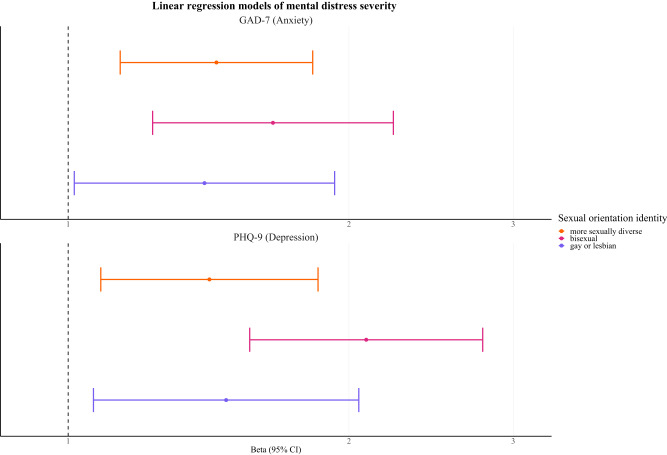
Linear regression models of mental distress severity per PHQ-9 and GAD-7

After adjusting for sociodemographic confounders, gay or lesbian (beta coefficient = 1.48; 95% CI = 1.06–2.05), bisexual (2.09; 1.57–2.78) and more sexually diverse (1.42; 1.08–1.85) individuals had a higher severity of depressive symptoms than their heterosexual counterparts. Similarly, we demonstrated higher anxiety symptoms severity in gay or lesbian (1.40; 1.02–1.93), bisexual (1.66; 1.23–2.23) and more sexually diverse individuals (1.44; 1.14–1.83). For detailed results, see Fig. 4 and Supplementary Table S5.

In the comparison of SM subgroups, with gay or lesbian individuals being the reference category, all results were consistent with a null effect. For detailed results, see Supplementary Table S5.

The results are expressed as adjusted beta coefficients with 95% confidence intervals. The models were adjusted for age, gender, education, work status, income level, relationship status and size of the region of residence. Heterosexual individuals were used as reference category.

### Treatment gap

We demonstrated broadly consistent levels of treatment gap in heterosexual and SM individuals fulfilling the criteria for any mental disorder (82.91%; 95% CI = 79.50–85.96 vs. 81.13%; 68.03–90.56). In SM individuals scoring positively for MDE, we detected a wider treatment gap than in heterosexual people (82.35%; 56.57–96.20 vs. 60.87%; 52.20–69.06). For AUDs, anxiety disorders and suicidal thoughts and behaviours, SM and heterosexual people had broadly consistent levels of treatment gap. For all results, see Table 2.

**Table 2.** Treatment gap prevalence for mental disorders established per M.I.N.I

	Heterosexual individuals	Sexual minority individuals
Any mental disorder	82.91 (79.50, 85.96)	81.13 (68.03, 90.56)
Alcohol use disorders	91.88 (87.97, 94.84)	92.59 (75.71, 99.09)
Major depressive disorder	60.87 (52.20, 69.06)	82.35 (56.57, 96.20)
Anxiety disorders	66.98 (60.26, 73.22)	69.57 (47.08, 86.79)
Suicidal thoughts and behaviours	72.46 (65.02, 79.07)	71.43 (51.33, 86.78)

The results are expressed as treatment gap prevalence rates with 95% confidence intervals.

### Sensitivity analyses

Using data from panel samples interviewed online and by telephone, the results were broadly consistent with the findings of the main analysis. Namely, SM individuals demonstrated generally higher rates of mental disorders and also higher symptom severity than their heterosexual counterparts. For detailed results, see Supplementary Table S1 and S6–S10.

## Discussion

### The main findings

In this first Czech nationally representative survey that included sexual identity and mental health measures, more than half of gay and lesbian respondents and a third of bisexual people met the criteria for at least one current mental disorder. We showed suicidal thoughts and behaviours in around 6% of heterosexual but in 25% of gay and lesbian and 23% of bisexual people. While bisexual people demonstrated higher rates of MDE and anxiety disorders than their gay or lesbian counterparts, when adjusting for sociodemographic characteristics, we did not detect differences between these two SM groups. The treatment gap in SM people having at least one mental disorder was very wide, reaching more than 80%, but in line with estimates for heterosexual people.

These findings underline the need for continued mental health monitoring of SM groups in Czechia, including in future public health surveys. Then, the detected mental health needs should stimulate service providers to introduce services tailored to SM people (Horne *et al.*, 2019). Finally, these findings should contribute to challenging the structural determinants that are most likely responsible for these outcomes, including the legal regulations and vocabulary used in public discourse.

### Mental health outcomes

Consistent with findings from abroad (Ploderl and Tremblay, 2015), we demonstrated substantially elevated rates of MDE and anxiety disorders in SM people. In gay and lesbian people, this is despite them being, on average, more educated and with higher income levels than their heterosexual counterparts: factors generally associated with better mental health outcomes. Indeed, existing research suggested that other structural factors such as stigma and minority stress might be responsible for worsened mental health outcomes in SM populations (Hatzenbuehler *et al.*, 2013; Pitoňák, 2017).

While approximately 6% of heterosexual people demonstrated suicidal thoughts and behaviours, a full one-quarter of gay and lesbian and nearly a quarter of bisexual people scored positively for these. Factors such as victimization, bullying or other forms of minority stress (Barnett *et al.*, 2019) may potentially thwart the sense of social belongingness among SM people and subsequently contribute to an elevated risk of suicidal ideation (Chu *et al.*, 2017; Joiner, 2005; Rogers *et al.*, 2021).

We showed that a full quarter of gay and lesbian respondents have met the criteria for AUDs, with the rates being considerably higher than in heterosexual people. However, a systematic review by Ploderl and Tremblay (2015) showed mixed evidence, with negative or near zero effect for SM men based on all studies but higher rates in most of higher quality studies using clinical diagnoses. The higher rates of AUDs among Czech SM people may be, in part, the consequence of the high normalization of alcohol drinking in Czech culture (Mravčík *et al.*, 2019), with alcohol consumption representing a highly prevalent dysfunctional coping strategy.

While most studies conducted abroad showed that bisexual individuals may have worse mental health compared with other SMs (Ross *et al.*, 2018), our evidence is mixed. Although we may only hypothesize, there are indications that the Czech gay and lesbian population may be affected by salient structural and interpersonal minority stressors compared with other SM groups. A pioneering Czech study on mental health in SM people showed that 45%, 40% and 39% of lesbian, gay and bisexual respondents felt discriminated against during the past 5 years, respectively (Pitoňák and Macháčková, 2023). The same study also demonstrated that gay individuals were most affected by physical assaults.

### Treatment gap

In four cross-sectional surveys conducted between 2017 and 2022, a consistently very wide treatment gap in the Czech general population was demonstrated, ranging from approximately 60% in people with MDE to around 90% in people with AUDs (Potočár *et al.*, 2024). The broadly consistent treatment gap in SM individuals and heterosexual people in the present study suggests that the treatment gap is uniformly wide in Czechia. Nevertheless, SM individuals may still face more of the same barriers as the heterosexual population and plausibly also specific ones.

For example, existing research suggests that minority stress undermines mental health in SM people by reducing access to health services ergo reducing the very benefits of care (Hatzenbuehler *et al.*, 2013). This may include barriers such as fear of disclosure due to expected discrimination from healthcare providers (McDermott *et al.*, 2018). In this context, it is important to note that currently there is no special lesbian, gay, bisexual and transgender+ (LGBT+) themed training required for mental healthcare providers available in Czechia, and the awareness of the minority stress framework is also relatively limited in the region (Pitoňák, 2017). In addition, specific healthcare services such as specialized support groups and ‘LGBT+ friendly’ therapeutic care providers (Steinke *et al.*, 2017) are currently underdeveloped in Czechia, and, if available, these are reliant on private and non-governmental resources.

### Systemic determinants of SM mental health and healthcare use in Czechia

Responsible institutions have, so far, failed to recognize the specific risk factors such as minority stress and stigma related to SM status (Hatzenbuehler *et al.*, 2013; Pitoňák, 2017). For example, the national suicide prevention plan by the Czech Ministry of Health does not include a single mention of SM, sexual and gender minority (SGM), or LGBT+-related terms. Available research demonstrates that either education strategies that aim to include LGBT+ topics and prevention of stressors such as antiqueer bullying (Hatzenbuehler, 2011) or legal changes such as an introduction of marriage equality (Raifman *et al.*, 2017) have measurable effects on reducing suicidality and minority stress among SM groups. Despite this, neither LGBT+ inclusive curriculum nor prevention of antiqueer bullying is a mandatory part of Czech education. Marriage equality law was first proposed in 2018, and it, unfortunately, became a gist of political anti-gender/anti-LGBT+ mobilization that hit not only Czechia. Anti-gender/anti-LGBT+ backlashes, including increases in physical violence, in neighbouring Poland, Slovakia and Hungary, seem to follow similar depreciation of SGM people from the site of politicians (Pitoňák, 2023). For instance, in 2022, 71% of Czech SGM respondents considered offensive statements by politicians to be ‘widespread’, contrasting with 43% in 2018 and 27% in 2012 (Pitoňák and Macháčková, 2023).

### Methodological considerations

Main strengths include using a considerably large, nationally representative sample and employment of a structured psycho-diagnostic instrument.

This study has some limitations. First, the number of SM individuals in our sample was considerably small, resulting in substantial uncertainty in some estimates. Second, the use of personal interviewing in household samples most likely contributed to the underestimation of the true number of SM individuals due to stigma-related concealment of their identities (Villarroel *et al.*, 2006). This is partially supported by a larger proportion of SM people present in our panel samples. However, there can be complex differences between samples obtained by different data collection methodologies (Potočár *et al.*, 2024): disentangling these should motivate future research. Third, we coded the individuals who chose the ‘other’ option on our sexual orientation measure as ‘more sexually diverse’; however, we do not know the precise composition of this group. Future research including more specific items in surveys is warranted. Fourth, due to the small sample size, we were not able to investigate the outcomes of transgender and non-binary individuals; thus, the mental health of gender minorities in Czechia remains largely unexplored. Last, due to financial constraints, we were not able to collect data on multiple important mental health outcomes, including psychotic and personality disorders. Furthermore, for the same reasons, we were unable to assess symptoms severity in conditions other than depression and anxiety.

## Conclusions

In this study, we reported results from the first Czech nationally representative survey to include sexual identity and mental health measures. We provide evidence that SM people are substantially more likely to have mental disorders than their heterosexual counterparts, with the disparities in rates for suicidal thoughts and behaviours being particularly striking. These inequalities in mental health remain largely unaddressed by responsible national institutions. Hence, it is time for systemic changes that would support the affirmative and non-pathologizing perspectives to provide not only better and more sensitive care to SM individuals but also to help expose and pull down the very fabric of structural stigma which is known as the driver of these health disparities.

## Supporting information

Pitoňák et al. supplementary material Pitoňák et al. supplementary material

## Data Availability

The respondents did not consent to have their data publicly available, but we will provide access to data upon a reasonable request (particularly replication). The complete analytical code is available at a dedicated GitHub repository: https://github.com/libpot/Mental_Health_Sex_Min_Czechia.

## Supplementary material

The supplementary material for this article can be found at https://doi.org/10.1017/S2045796024000210.
