# Supplementary material for: Mental health and help-seeking in Czech sexual minorities: a nationally representative cross-sectional study
Source: Epidemiol Psychiatr Sci. 2024 Mar 21;33:e16. doi: 10.1017/S2045796024000210 (PMC11022263; doi:10.1017/S2045796024000210)
Supplement: Pitoňák et al. supplementary material [file S2045796024000210sup001.docx]

# Supplementary Methods

## Deviations from the protocol

First, to ensure that the confidence intervals for treatment gap prevalence do not exceed 100%, we used the exact binomial Clopper-Person method to estimate the confidence intervals.

Second, the distribution of scores on PHQ-9 and GAD-7 was heavily right skewed, leading to violation of the homoscedasticity assumption of linear regression. Thus, we log-transformed the PHQ-9 and GAD-7 scores before their inclusion to the models and estimated robust (sandwich) standard errors.

Last, to avoid excessive uncertainty due very small number of individuals, we removed the models focused on the likelihood of seeking help for mental disorders in SM people.

# Supplementary Results

Table S1 Descriptive statistics of the panel sample

|  | **Sexual orientation identity** | | | |
| --- | --- | --- | --- | --- |
|  | Heterosexual | Gay or lesbian | Bisexual | More sexually diverse |
| Sample size, n (%) | 3903 (91.9%) | 69 (1.62%) | 147 (3.46%) | 129 (3.04%) |
| Sex, n (%) |  | | | |
| female | 2035 (52.14) | 10 (14.49) | 92 (62.59) | 79 (61.24) |
| male | 1868 (47.86) | 59 (85.51) | 55 (37.41) | 50 (38.76) |
| Gender, n (%) |  | | | |
| women | 2028 (51.96) | 12 (17.39) | 88 (59.86) | 74 (57.36) |
| men | 1870 (47.91) | 57 (82.61) | 58 (39.46) | 45 (34.88) |
| non-binary | 2 (0.05) | 0 (0) | 1 (0.68) | 8 (6.2) |
| transgender | 3 (0.08) | 0 (0) | 0 (0) | 2 (1.55) |
| Age, mean (SD) | 48.83 (16.07) | 42.83 (15.7) | 40.43 (18.36) | 49.95 (18.68) |
| Relationship status, n (%) |  | | | |
| married/in a relationship, living together | 2370 (60.72) | 29 (42.03) | 81 (55.1) | 67 (51.94) |
| married/in a relationship, living apart | 179 (4.59) | 4 (5.8) | 17 (11.56) | 7 (5.43) |
| single | 611 (15.65) | 31 (44.93) | 36 (24.49) | 26 (20.16) |
| divorced | 550 (14.09) | 2 (2.9) | 10 (6.8) | 20 (15.5) |
| widowed | 193 (4.94) | 3 (4.35) | 3 (2.04) | 9 (6.98) |
| Level of education, n (%) |  | | | |
| primary | 503 (12.89) | 7 (10.14) | 26 (17.69) | 19 (14.73) |
| lower secondary | 1294 (33.15) | 18 (26.09) | 52 (35.37) | 56 (43.41) |
| upper secondary | 1398 (35.82) | 26 (37.68) | 53 (36.05) | 39 (30.23) |
| university | 708 (18.14) | 18 (26.09) | 16 (10.88) | 15 (11.63) |
| Work status, n (%) |  | | | |
| employed | 2100 (53.8) | 37 (53.62) | 65 (44.22) | 46 (35.66) |
| unemployed | 117 (3) | 1 (1.45) | 8 (5.44) | 2 (1.55) |
| self-employed | 231 (5.92) | 4 (5.8) | 10 (6.8) | 9 (6.98) |
| student | 162 (4.15) | 7 (10.14) | 24 (16.33) | 16 (12.4) |
| retired | 850 (21.78) | 11 (15.94) | 23 (15.65) | 41 (31.78) |
| parental leave | 179 (4.59) | 2 (2.9) | 10 (6.8) | 3 (2.33) |
| other | 264 (6.76) | 7 (10.14) | 7 (4.76) | 12 (9.3) |
| Income category, n (%) |  | | | |
| 0-9k CZK | 343 (8.79) | 5 (7.25) | 22 (14.97) | 12 (9.3) |
| 10-19k CZK | 1041 (26.67) | 14 (20.29) | 46 (31.29) | 50 (38.76) |
| 20-29k CZK | 1136 (29.11) | 12 (17.39) | 46 (31.29) | 31 (24.03) |
| 30-39k CZK | 576 (14.76) | 16 (23.19) | 11 (7.48) | 5 (3.88) |
| 40-49k CZK | 247 (6.33) | 8 (11.59) | 4 (2.72) | 3 (2.33) |
| 50+k CZK | 157 (4.02) | 5 (7.25) | 3 (2.04) | 1 (0.78) |
| decided not to disclose | 403 (10.33) | 9 (13.04) | 15 (10.2) | 27 (20.93) |
| Size of the region of residence, n (%) |  | | | |
| >5000 | 1507 (38.61) | 17 (24.64) | 60 (40.82) | 56 (43.41) |
| 5000-19999 | 723 (18.52) | 6 (8.7) | 24 (16.33) | 21 (16.28) |
| 20 000-99 999 | 831 (21.29) | 16 (23.19) | 26 (17.69) | 32 (24.81) |
| 100 000+ | 842 (21.57) | 30 (43.48) | 37 (25.17) | 20 (15.5) |
| Mode of interviewing, n (%) |  |  |  |  |
| online | 2948 (75.53) | 51 (73.91) | 132 (89.8) | 117 (90.7) |
| telephone | 955 (24.47) | 18 (26.09) | 15 (10.2) | 12 (9.3) |

The results are expressed as either absolute numbers (n) with percent proportions (%) or averages with standard deviations (SD).

Table S2 Prevalence rates of mental disorders per M.I.N.I. in the household sample

|  | Any mental disorder | Alcohol use disorders | Major depressive episode | Anxiety disorders | Suicidal thoughts and behaviors |
| --- | --- | --- | --- | --- | --- |
| Heterosexual | 18.85 (17.43, 20.28) | 9.29 (8.24, 10.34) | 4.73 (3.96, 5.5) | 7.37 (6.42, 8.32) | 5.73 (4.88, 6.57) |
| Gay or lesbian | 52.27 (36.91, 67.63) | 25 (11.68, 38.32) | 11.36 (3.79, 24.56) | 15.91 (4.66, 27.16) | 25 (11.68, 38.32) |
| Bisexual | 33.33 (19.5, 47.17) | 16.67 (5.73, 27.6) | 16.67 (5.73, 27.6) | 20.83 (8.92, 32.75) | 22.92 (10.58, 35.25) |
| More sexually diverse | 25.93 (13.85, 38) | 14.81 (5.03, 24.6) | 7.41 (2.06, 17.89) | 11.11 (2.45, 19.77) | 11.11 (2.45, 19.77) |

The results are expressed as prevalence rates with 95% confidence intervals.

Table S3 Relative risk of mental disorder occurrence per M.I.N.I. in the household sample

|  | Ref. cat.: heterosexual individuals | | | | | Ref. cat.: gay or lesbian individuals | | | | |
| --- | --- | --- | --- | --- | --- | --- | --- | --- | --- | --- |
|  | Any mental disorder | Major depressive episode | Anxiety disorders | Alcohol use disorders | Suicidal thoughts and behaviors | Any mental disorder | Major depressive episode | Anxiety disorders | Alcohol use disorders | Suicidal thoughts and behaviors |
| Gay or lesbian | 3.51 (1.83, 6.76) | 2.64 (0.83, 6.92) | 2.18 (0.83, 5.03) | 1.66 (0.73, 3.52) | 4.49 (1.99, 9.53) | NA | NA | NA | NA | NA |
| Bisexual | 1.85 (0.96, 3.45) | 3.55 (1.42, 7.89) | 3.21 (1.44, 6.62) | 1.63 (0.68, 3.52) | 4.76 (2.18, 9.7) | 0.53 (0.21, 1.28) | 1.35 (0.37, 5.29) | 1.47 (0.48, 4.75) | 0.98 (0.32, 2.96) | 1.06 (0.37, 3.02) |
| More sexually diverse | 0.89 (0.42, 1.73) | 1.29 (0.37, 3.43) | 1.07 (0.38, 2.51) | 0.8 (0.29, 1.91) | 1.01 (0.31, 2.59) | 0.25 (0.1, 0.63) | 0.49 (0.1, 2.17) | 0.49 (0.14, 1.7) | 0.48 (0.14, 1.51) | 0.23 (0.06, 0.74) |

The results are expressed as adjusted odds ratios with 95% confidence intervals. The models were adjusted for age, gender, education, work status, income level, relationship status, and size of the region of residence. “Ref. cat.: heterosexual” indicates that heterosexual individuals were used as reference category. “Ref. cat.: gay or lesbian” indicates that gay or lesbian individuals were used as reference category.

Table S4 Mean PHQ-9 and GAD-7 scores in the household sample

|  | PHQ-9 | GAD-7 |
| --- | --- | --- |
| Heterosexual | 2.96 (2.81, 3.11) | 1.97 (1.85, 2.08) |
| Gay or lesbian | 4.68 (2.95, 6.42) | 3.5 (1.98, 5.02) |
| Bisexual | 7.12 (5.07, 9.18) | 4.63 (3.05, 6.2) |
| More sexually diverse | 5.17 (3.38, 6.95) | 3.7 (2.29, 5.11) |

The results are expressed as averages with 95% confidence intervals.

Table S5 Linear regression models of mental distress severity per PHQ-9 and GAD-7 in the household sample

|  | Ref. cat.: heterosexual | | Ref. cat.: gay or lesbian | |
| --- | --- | --- | --- | --- |
|  | PHQ-9 | GAD-7 | PHQ-9 | GAD-7 |
| Heterosexual | NA | NA | NA | NA |
| Gay or lesbian | 1.48 (1.06, 2.05) | 1.4 (1.02, 1.93) | NA | NA |
| Bisexual | 2.09 (1.57, 2.78) | 1.66 (1.23, 2.23) | 1.41 (0.92, 2.18) | 1.18 (0.77, 1.83) |
| More sexually diverse | 1.42 (1.08, 1.85) | 1.44 (1.14, 1.83) | 0.96 (0.64, 1.45) | 1.03 (0.7, 1.52) |

The results are expressed as adjusted beta coefficients with 95% confidence intervals. The models were adjusted for age, gender, education, work status, income level, relationship status, and size of the region of residence. “Ref. cat.: heterosexual” indicates that heterosexual individuals were used as reference category. “Ref. cat.: gay or lesbian” indicates that gay or lesbian individuals were used as reference category.

Table S6 Prevalence rates of mental disorders per M.I.N.I. in the panel sample

|  | Any mental disorder | Alcohol use disorders | Major depressive episode | Anxiety disorders | Suicidal thoughts and behaviors |
| --- | --- | --- | --- | --- | --- |
| Heterosexual | 31.87 (30.41, 33.34) | 12.22 (11.19, 13.25) | 11.35 (10.35, 12.35) | 15.14 (14.02, 16.27) | 13.02 (11.96, 14.07) |
| Gay or lesbian | 37.68 (25.95, 49.41) | 15.94 (7.08, 24.8) | 11.59 (3.85, 19.34) | 21.74 (11.76, 31.72) | 20.29 (10.56, 30.02) |
| Bisexual | 63.27 (55.38, 71.15) | 27.89 (20.56, 35.23) | 35.37 (27.55, 43.19) | 36.73 (28.85, 44.62) | 30.61 (23.07, 38.15) |
| More sexually diverse | 35.66 (27.28, 44.04) | 13.95 (7.89, 20.01) | 11.63 (6.02, 17.23) | 18.6 (11.8, 25.41) | 17.83 (11.14, 24.52) |

The results are expressed as prevalence rates with 95% confidence intervals.

Table S7 Relative risk of mental disorder occurrence per M.I.N.I. in the panel sample

|  | Ref. cat.: heterosexual individuals | | | | | Ref. cat.: gay or lesbian individuals | | | | |
| --- | --- | --- | --- | --- | --- | --- | --- | --- | --- | --- |
|  | Any mental disorder | Major depressive episode | Anxiety disorders | Alcohol use disorders | Suicidal thoughts and behaviors | Any mental disorder | Major depressive episode | Anxiety disorders | Alcohol use disorders | Suicidal thoughts and behaviors |
| Gay or lesbian | 1.11 (0.66, 1.85) | 1.06 (0.45, 2.2) | 1.79 (0.94, 3.22) | 0.89 (0.43, 1.69) | 1.77 (0.92, 3.21) | NA | NA | NA | NA | NA |
| Bisexual | 2.83 (1.97, 4.09) | 3.13 (2.11, 4.59) | 2.41 (1.64, 3.52) | 2.21 (1.47, 3.29) | 2.17 (1.46, 3.18) | 2.54 (1.37, 4.77) | 2.94 (1.3, 7.39) | 1.35 (0.68, 2.8) | 2.48 (1.17, 5.61) | 1.22 (0.61, 2.58) |
| More sexually diverse | 0.99 (0.65, 1.49) | 0.85 (0.45, 1.49) | 0.95 (0.55, 1.55) | 1.15 (0.62, 2.01) | 1.01 (0.58, 1.67) | 0.89 (0.46, 1.71) | 0.8 (0.31, 2.19) | 0.53 (0.24, 1.17) | 1.29 (0.54, 3.19) | 0.57 (0.26, 1.29) |

The results are expressed as adjusted odds ratios with 95% confidence intervals. The models were adjusted for age, gender, education, work status, income level, relationship status, size of the region of residence, and mode of interviewing (telephone vs. online). “Ref. cat.: heterosexual” indicates that heterosexual individuals were used as reference category. “Ref. cat.: gay or lesbian” indicates that gay or lesbian individuals were used as reference category.

Table S8 Mean PHQ-9 and GAD-7 scores in the panel sample

|  | PHQ-9 | GAD-7 |
| --- | --- | --- |
| Heterosexual | 5.16 (5, 5.33) | 3.28 (3.15, 3.41) |
| Gay or lesbian | 5.25 (3.93, 6.56) | 3.61 (2.58, 4.64) |
| Bisexual | 9.44 (8.38, 10.49) | 6.88 (5.88, 7.88) |
| More sexually diverse | 5.86 (4.83, 6.89) | 3.91 (3.06, 4.77) |

The results are expressed as averages with 95% confidence intervals.

Table S9 Linear regression models of mental distress severity per PHQ-9 and GAD-7 in the panel sample

|  | Ref. cat.: heterosexual | | Ref. cat.: gay or lesbian | |
| --- | --- | --- | --- | --- |
|  | PHQ-9 | GAD-7 | PHQ-9 | GAD-7 |
| Heterosexual | NA | NA | NA | NA |
| Gay or lesbian | 0.96 (0.78, 1.17) | 1.04 (0.84, 1.29) | NA | NA |
| Bisexual | 1.51 (1.34, 1.7) | 1.54 (1.34, 1.77) | 1.58 (1.25, 1.99) | 1.49 (1.15, 1.91) |
| More sexually diverse | 0.93 (0.8, 1.09) | 0.99 (0.85, 1.16) | 0.97 (0.75, 1.26) | 0.95 (0.73, 1.24) |

The results are expressed as adjusted beta coefficients with 95% confidence intervals. The models were adjusted for age, gender, education, work status, income level, relationship status, size of the region of residence, and mode of interviewing (telephone vs. online). “Ref. cat.: heterosexual” indicates that heterosexual individuals were used as reference category. “Ref. cat.: gay or lesbian” indicates that gay or lesbian individuals were used as reference category.

Table S10 Treatment gap prevalence for mental disorders established per M.I.N.I. in the panel sample

|  | Heterosexual individuals | Sexual minority individuals |
| --- | --- | --- |
| Any mental disorder | 75.64 (73.16, 78.01) | 65.45 (57.67, 72.67) |
| Alcohol use disorders | 84.49 (80.92, 87.62) | 70 (57.87, 80.38) |
| Major depressive disorder | 63.43 (58.76, 67.93) | 60 (48.04, 71.15) |
| Anxiety disorders | 65.82 (61.84, 69.64) | 51.61 (41.01, 62.11) |
| Suicidal thoughts and behaviors | 66.14 (61.84, 70.25) | 60.98 (49.57, 71.56) |

The results are expressed as treatment gap prevalence rates with 95% confidence intervals.
